# 

**Published:** 1869-02-15

**Authors:** 


VOL. XXVI. NUMBER 4.

THE

### CHICAGO

### Medical Journal.

PUBLISHED SEMI-MONTHLY.

J. ADAMS ALLEN, M. D., LL. D.,
*EDITOR.*

WALTER HAY, A. M., M. D.,
*ASSOCIATE EDITOR.*

FEBRUARY 15, 1869.

CHICAGO:
HORTON & LEONARD, STEAM BOOK AND JOB PRINTERS,
104 & 106 Randolph Street.

